# Complete Mitochondrial Genome and Phylogenetic Analysis of the Chungtien Schizothoracin (*Ptychobarbus chungtienensis*)

**DOI:** 10.3390/cimb48090888

**Published:** 2026-08-31

**Authors:** Yuwei Qian, Ruoshan Ma, Shiguang Ma, Zhen Wang, Weidong Deng, Zhendong Gao, Bo Wang

**Affiliations:** 1 Yunnan Provincial Key Laboratory of Animal Science and Feed, Faculty of Animal Science and Technology, Yunnan Agricultural University, Kunming 650201, China; 2 Diqing Tibetan Autonomous Prefecture Institute of Animal Husbandry and Veterinary Science, Shangri-La 674499, China

**Keywords:** *Ptychobarbus chungtienensis*, mitochondrial genome, phylogenetic analysis, schizothoracinae

## Abstract

Background: The Chungtien schizothoracin (*Ptychobarbus chungtienensis*) is a threatened freshwater fish endemic to the Qinghai–Tibet Plateau and adjacent high-altitude regions of northwestern Yunnan, China. Although a complete mitochondrial genome of *P. chungtienensis* has been previously reported, direct comparison with a mitogenome generated using high-accuracy long-read sequencing can provide additional information on mitochondrial genome structure and sequence variation. This study aimed to assemble and annotate a complete mitogenome of *P. chungtienensis* using PacBio HiFi sequencing and to compare its mitogenomic characteristics with previously published *Ptychobarbus* mitogenomes. Methods: High-molecular-weight genomic DNA from a single specimen was sequenced using PacBio HiFi long-read technology. The mitochondrial genome was assembled using MitoHiFi, annotated using MitoFinder followed by manual curation, and compared with previously published *Ptychobarbus* mitogenomes. Phylogenetic relationships were evaluated using maximum-likelihood analysis with expanded taxon sampling, and selection pressure on the 13 mitochondrial protein-coding genes was assessed using dN/dS-based branch and branch-site models. Results: The assembled mitogenome is 16,583 bp in length and contains the typical 37 mitochondrial genes, including 13 protein-coding genes, 22 tRNA genes, and 2 rRNA genes, together with a control region and the origin of light-strand replication (O*_L_*). The overall A + T content was 54.97%. Direct comparison with the previously reported 16,970 bp mitogenome showed that the 387 bp length difference was concentrated in non-coding regions, particularly the control region and the tRNA-Thr–tRNA-Pro intergenic region. Phylogenetic analysis based on 22 complete mitogenomes placed the newly assembled *P. chungtienensis* sequence in a strongly supported mitochondrial clade with *Schizothorax macropogon* (bootstrap = 100%), whereas the previously reported *P. chungtienensis* sequence clustered with *P. kaznakovi* (bootstrap = 100%), indicating that the two *P. chungtienensis* records represent distinct mitochondrial lineages. The dN/dS values of all 13 mitochondrial protein-coding genes were below 1, and neither branch nor branch–site analyses detected significant evidence of lineage-specific positive selection. Conclusions: This long-read-based mitogenome provides a high-quality genomic resource for *P. chungtienensis* and reveals substantial mitochondrial sequence and lineage variation among available records. These results provide a basis for comparative mitogenomic and conservation genetic studies while also indicating that species-level phylogenetic relationships and high-altitude adaptation should not be inferred from mitochondrial data alone.

## 1. Introduction

The Chungtien schizothoracin, *Ptychobarbus chungtienensis*, is an endemic freshwater fish restricted mainly to the Zhongdian Plateau and surrounding waters in northwestern Yunnan, China, where it inhabits high-altitude aquatic environments [1,2,3]. Its habitats are characterized by low temperature, reduced oxygen availability, and marked seasonal environmental variation, making the species relevant to studies of the evolution and ecology of plateau fishes [1,2,3]. The species has been recognized as threatened and is of considerable conservation concern because of its restricted distribution [4]. Despite its ecological and conservation significance, mitochondrial genomic resources for *P. chungtienensis* remain limited, and only a small number of mitogenomic studies are available for direct comparison within *Ptychobarbus*. Schizothoracine fishes provide an important system for investigating the evolutionary history of fishes inhabiting the Qinghai–Tibet Plateau and adjacent high-altitude regions. Previous comparative mitogenomic studies have reported signatures of selection in mitochondrial protein-coding genes of some schizothoracine fishes and proposed their possible association with high-altitude environments [5,6]. Transcriptomic and nuclear genomic studies have also identified multiple biological processes potentially associated with adaptation to plateau environments [7,8]. These studies provide a broader evolutionary context for mitochondrial genomic analyses but do not imply that mitogenomic variation alone is sufficient to explain high-altitude adaptation.

Vertebrate mitochondrial genomes are compact, maternally inherited molecules that encode key components of oxidative phosphorylation together with mitochondrial rRNAs and tRNAs [9,10]. Although mitochondrial gene content is generally conserved among vertebrates, genome length and non-coding regions, particularly the control region, can vary because of substitutions, insertions/deletions, and repetitive sequences [10,11]. A complete mitochondrial genome of *P. chungtienensis* was previously reported by Qiu and Chen in 2016 using a PCR-based sequencing strategy, with a total length of 16,970 bp [1]. This study established an important mitochondrial reference for the species, but direct comparison with independently generated long-read data may help evaluate sequence and structural variation, particularly in repeat-rich non-coding regions. PacBio HiFi sequencing generates highly accurate long reads and is well suited for resolving repetitive and structurally variable genomic regions [12,13,14]. For mitochondrial genomes, these properties facilitate direct assessment of genome length, sequence differences, and repetitive elements in regions that may be difficult to characterize using short reads or PCR-based approaches. Long-read mitogenome studies have also shown that repetitive structures and length variation may be underestimated in existing mitochondrial references [11,14]. Therefore, direct comparison of independently generated mitogenomes can provide useful information on structural and sequence variation.

In the present study, we assembled and annotated the complete mitochondrial genome of *P. chungtienensis* using PacBio HiFi sequencing data. We further compared its genome organization, nucleotide composition, non-coding regions, and sequence divergence with previously published *Ptychobarbus* mitogenomes, with particular attention to the previously reported *P. chungtienensis* sequence. Phylogenetic relationships were re-evaluated using expanded taxon sampling that included available *Ptychobarbus* mitogenomes and closely related schizothoracine fishes, with statistical support assessed for the resulting mitochondrial lineages. In addition, selection pressure on the 13 mitochondrial protein-coding genes was evaluated to characterize patterns of mitochondrial sequence evolution. Together, these analyses were designed to provide a more complete comparative mitogenomic framework for *P. chungtienensis* while avoiding species-level evolutionary inferences based solely on mitochondrial data.

## 2. Materials and Methods

### 2.1. Sample Collection and DNA Preparation

**Figure 1:**
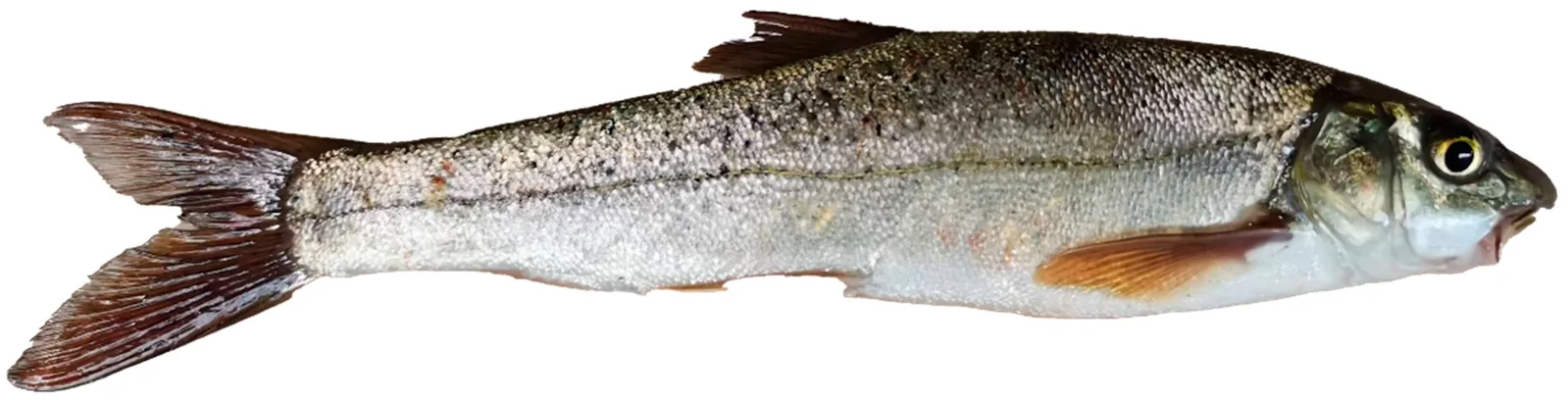
Specimen of the Chungtien schizothoracin (*Ptychobarbus chungtienensis*) collected from the Luoji River, Yunnan Province, China. Photograph taken by Zhen Wang on 15 April 2022.

The Chungtien schizothoracin (*Ptychobarbus chungtienensis*) specimen used in this study was collected during an aquatic biodiversity survey conducted by the Diqing Tibetan Autonomous Prefecture Institute of Animal Husbandry and Veterinary Science in the Luoji River, Shangri-La, Diqing Tibetan Autonomous Prefecture, Yunnan Province, China (100.276754° E, 27.791412° N). The specimen was approximately 4–6 months old (Figure 1). High-molecular-weight genomic DNA used for PacBio HiFi sequencing was extracted from fresh tissue from this single individual using a modified CTAB method [15]. No DNA from different tissues or individuals was pooled for mitochondrial genome sequencing. The voucher specimen was deposited at the Yunnan Provincial Key Laboratory of Animal Science and Feed under the identifier CCY-202312001 (contact: Zhendong Gao, Zander_Gao@163.com).

DNA concentration and quality were assessed using a NanoDrop 2000 spectrophotometer, a Qubit 3.0 fluorometer (Thermo Fisher Scientific, Waltham, MA, USA), and 0.8% agarose gel electrophoresis. Only high-quality DNA meeting the requirements for PacBio HiFi library construction was used for sequencing.

### 2.2. PacBio HiFi Library Construction and Sequencing

High-molecular-weight genomic DNA was fragmented to approximately 15 kb using a g-TUBE device (Covaris, Woburn, MA, USA), followed by library construction using the SMRTbell Express Template Prep Kit 2.0 (Pacific Biosciences, Menlo Park, CA, USA). Approximately 15 μg of genomic DNA was subjected to DNA damage repair and end-repair reactions, followed by adapter ligation. The ligation reaction was performed at 20 °C for 15 h, after which the library was purified using AMPure PB magnetic beads.

Library concentration and fragment-size distribution were assessed using a FEMTO Pulse system (Agilent Technologies, Santa Clara, CA, USA) and a Qubit 3.0 fluorometer. Size selection was performed using the BluePippin system (Sage Science, Beverly, MA, USA) to remove SMRTbell fragments shorter than 15 kb. The purified library was subsequently evaluated using FEMTO Pulse and the Qubit dsDNA HS Assay Kit (Thermo Fisher Scientific, Waltham, MA, USA) and then prepared for sequencing with sequencing primers and Sequel II DNA polymerase.

The final library was sequenced at a loading concentration of 120 pM on the PacBio Sequel II platform with a 30-h sequencing run. Sequencing was performed by Wuhan Frasergen Gene Biotechnology Co., Ltd., Wuhan, Hubei, China.

### 2.3. Mitochondrial Genome Assembly and Annotation

PacBio HiFi reads were used to assemble the mitochondrial genome of *Ptychobarbus chungtienensis* using MitoHiFi v3.2.1 [14]. The resulting mitochondrial sequence was subsequently annotated using MitoFinder v1.4.2, the previously published *P. chungtienensis* mitogenome (KY012741; NC_034230.1). Functional annotation of the mitochondrial genome was performed using Mitofinder [16]. The mitochondrial genome map was visualized using CGView v2.0 [17].

The automated annotation was manually inspected and curated by comparison with the previously published *P. chungtienensis* mitogenome and other available *Ptychobarbus* mitochondrial genomes. Particular attention was given to non-coding regions that were not explicitly identified during automated annotation. The control region (CR) was identified between tRNA-Pro and tRNA-Phe, whereas the origin of light-strand replication (O*_L_*) was identified within the WANCY tRNA cluster between tRNA-Asn and tRNA-Cys. Gene boundaries, transcriptional orientation, and incomplete termination codons were also manually checked.

The circular mitochondrial genome map was generated using Proksee (https://proksee.ca). Protein-coding genes, tRNA genes, rRNA genes, the CR, O*_L_*, GC content, and GC skew were displayed according to their genomic positions and transcriptional orientations.

### 2.4. Comparative Mitogenomic Analysis

The newly assembled *P. chungtienensis* mitogenome was compared with available complete mitochondrial genomes of *Ptychobarbus*, including the previously reported *P. chungtienensis* mitogenome (KY012741.1/NC_034230.1), *P. dipogon* (KF597526.1), and *P. kaznakovi* (KM268050.1). Genome length, nucleotide composition, A + T content, AT-skew, GC-skew, gene organization, and major non-coding regions were compared among the four mitogenomes. AT-skew and GC-skew were calculated as (A − T)/(A + T) and (G − C)/(G + C), respectively.

To characterize the 387 bp length difference between the present 16,583 bp mitogenome and the previously reported 16,970 bp *P. chungtienensis* mitogenome, the two complete sequences were directly compared using NUCmer v4.0.0rc1 [18]. Alignment coordinates and nucleotide differences were examined using delta-filter, show-coords, and show-snps. The control regions of the two mitogenomes were additionally aligned using MAFFT to examine sequence and structural variation. Tandem repeats within the control regions were detected using Tandem Repeats Finder (TRF) v4.09 [19] with the parameters 2, 7, 7, 80, 10, 50, and 500.

For assessment of sequence divergence within *Ptychobarbus*, the four complete mitochondrial genome sequences were aligned using MAFFT v7.526 [20]. Pairwise nucleotide identity was calculated from aligned positions containing unambiguous nucleotides in both sequences, with gaps and ambiguous bases excluded. Pairwise p-distance was calculated as the proportion of nucleotide mismatches among comparable aligned sites.

### 2.5. Phylogenetic Analysis

To evaluate the mitochondrial lineage relationships of *P. chungtienensis*, phylogenetic analysis was performed using 22 complete mitochondrial genome sequences, including the newly assembled *P. chungtienensis* mitogenome (PQ766596.1), the previously reported *P. chungtienensis* mitogenome (KY012741.1), *P. dipogon* (KF597526.1), *P. kaznakovi* (KM268050.1), and representative cyprinid fishes listed in Table A1. *Puntigrus tetrazona* (NC_010110.1) was selected as the outgroup.

Complete mitochondrial genome sequences were aligned using MAFFT [20]. The best-fitting nucleotide substitution model was selected using ModelFinder implemented in IQ-TREE v3.1.3 [21] according to the Bayesian information criterion (BIC). Maximum-likelihood phylogenetic reconstruction was subsequently performed using IQ-TREE under the TIM2 + F + I + R3 model. Node support was assessed using 1000 standard nonparametric bootstrap replicates. The resulting tree was rooted using *P. tetrazona* as the outgroup.

### 2.6. Selection Pressure Analysis

To assess selective pressure on mitochondrial protein-coding genes, the 13 mitochondrial PCGs (*ND1*, *ND2*, *COX1*, *COX2*, *ATP8*, *ATP6*, *COX3*, *ND3*, *ND4L*, *ND4*, *ND5*, *ND6*, and *CYTB*) were extracted from the mitochondrial genomes included in the phylogenetic analysis and analyzed separately. Protein-coding sequences were translated using the vertebrate mitochondrial genetic code, and amino-acid sequences of each gene were aligned using MAFFT [20]. The amino-acid alignments were subsequently back-translated to generate codon-based nucleotide alignments for selection-pressure analysis.

The ratio of nonsynonymous to synonymous substitution rates (dN/dS, ω) was estimated using the codeml program implemented in PAML v4.10.9 [22]. The maximum-likelihood topology inferred from the complete mitochondrial genomes was used for codeml analyses, with the branch leading to the newly sequenced *P. chungtienensis* mitogenome specified as the foreground branch. The vertebrate mitochondrial genetic code was specified in codeml, and codon frequencies were estimated using the F3 × 4 model.

Three complementary codon models were applied to each of the 13 PCGs. First, the one-ratio model (M0) was used to estimate the average ω value across all branches. Second, the two-ratio branch model was applied to estimate separate ω values for the foreground and background branches. Third, branch-site model A was used to test for positive selection affecting a subset of codon sites along the foreground lineage. The branch-site alternative model allowing ω > 1 was compared with the corresponding null model in which the positively selected class was constrained to ω = 1.

Likelihood-ratio tests (LRTs) were used to compare the two-ratio branch model with M0 and the branch-site alternative model with its corresponding null model. The test statistic was calculated as 2ΔlnL = 2(lnL1 − lnL0) and evaluated using a chi-square distribution with one degree of freedom. Statistical significance was defined as *p* < 0.05.

## 3. Results

### 3.1. Mitochondrial Genome Organization and Composition

**Figure 2:**
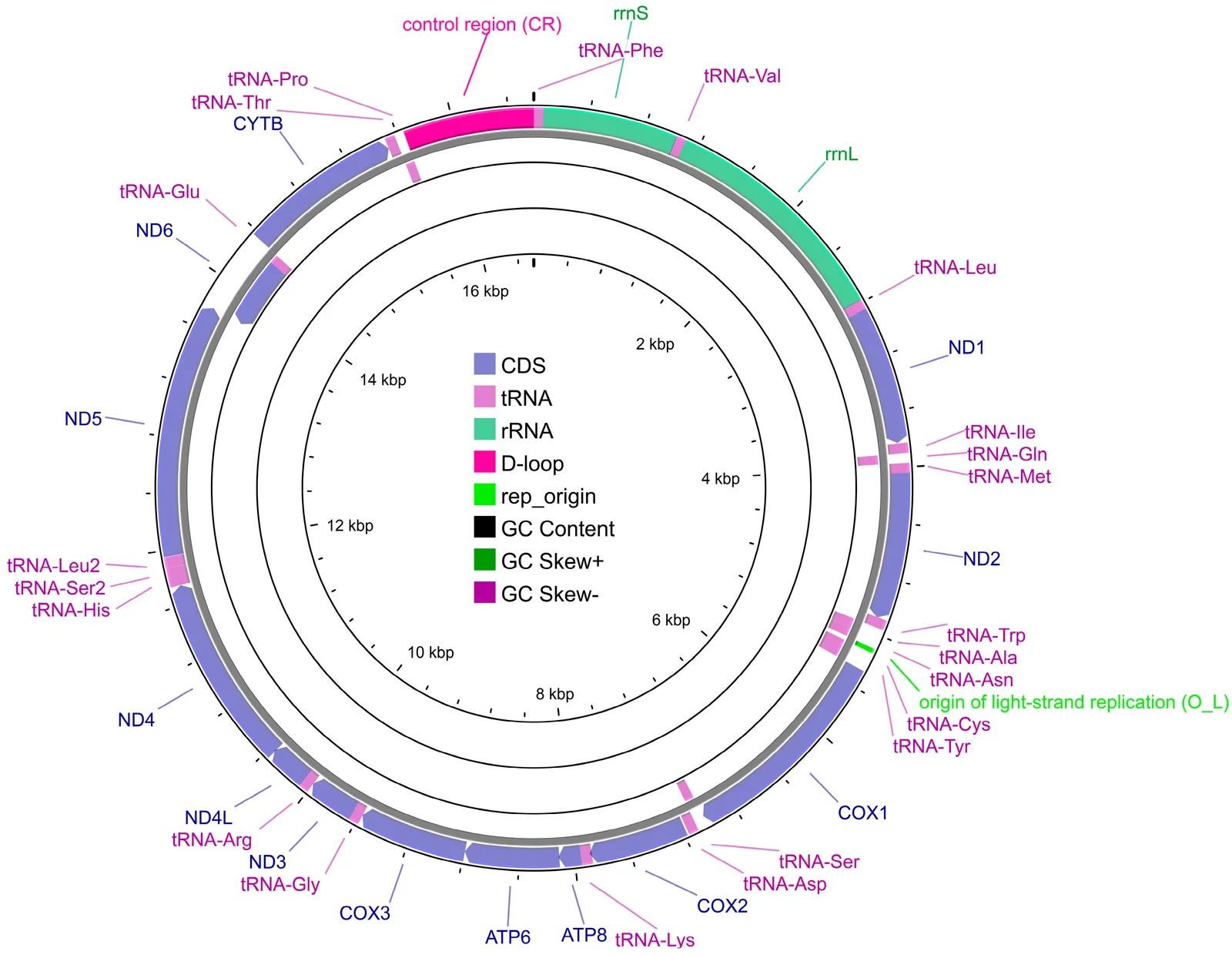
Circular map of the mitochondrial genome of *Ptychobarbus chungtienensis*. Protein-coding genes, tRNA genes, rRNA genes, the control region (CR), and the origin of light-strand replication (O*_L_*) are shown according to their genomic positions. Arrow directions indicate the transcriptional orientation of genes on the heavy (H) and light (L) strands. The inner tracks represent GC content and GC skew.

The complete mitochondrial genome of *Ptychobarbus chungtienensis* was 16,583 bp in length and contained the typical vertebrate mitochondrial gene complement of 37 genes, including 13 protein-coding genes (PCGs), 22 transfer RNA (tRNA) genes, and two ribosomal RNA (rRNA) genes. A control region (CR) and the origin of light-strand replication (O*_L_*) were also identified (Figure 2). The 13 PCGs were *ND1*, *ND2*, *COX1*, *COX2*, *ATP8*, *ATP6*, *COX3*, *ND3*, *ND4L*, *ND4*, *ND5*, *ND6*, and *CYTB*. The two rRNA genes were 12S rRNA and 16S rRNA, and the 22 tRNA genes corresponded to the 20 standard amino acids.

Most mitochondrial genes were encoded on the heavy strand (H-strand), whereas *ND6* and eight tRNA genes—tRNA-Gln, tRNA-Ala, tRNA-Asn, tRNA-Cys, tRNA-Tyr, tRNA-Ser, tRNA-Glu, and tRNA-Pro—were encoded on the light strand (L-strand). The CR was located between tRNA-Pro and tRNA-Phe at positions 15,650–16,583 and was 934 bp in length. O*_L_* was located within the WANCY tRNA cluster between tRNA-Asn and tRNA-Cys at positions 5303–5335 and was 33 bp in length.

The nucleotide composition of the mitogenome was 29.68% A, 25.28% T, 27.20% C, and 17.84% G, with an overall A + T content of 54.97%. The AT-skew and GC-skew were 0.080 and −0.208, respectively.

### 3.2. Comparative Mitogenomic Analysis of Ptychobarbus

Comparison of the four available *Ptychobarbus* mitochondrial genome records included in this study revealed variation in genome length, nucleotide composition, nucleotide skews, and non-coding-region characteristics (Table 1). The complete mitochondrial genomes ranged from 16,583 bp in the present *P. chungtienensis* assembly to 16,970 bp in the previously reported *P. chungtienensis* mitogenome. Despite these differences, all four mitochondrial genomes exhibited a conserved gene complement and gene arrangement, consisting of 13 protein-coding genes, 22 tRNA genes, and two rRNA genes. The overall A + T contents ranged from 53.76% to 54.97%. All mitogenomes showed positive AT-skew values and negative GC-skew values, indicating a conserved strand-specific nucleotide composition bias among *Ptychobarbus* mitochondrial genomes.

The previously published *P. chungtienensis* mitogenome (KY012741.1/NC_034230.1) was reported to contain a control region (D-loop) of 1161 bp. The approximately 1162 bp value shown in Table 1 corresponds to the boundary definition used for direct sequence comparison in the present study.

Direct comparison between the present 16,583 bp mitogenome and the previously reported 16,970 bp *P. chungtienensis* mitogenome revealed a total length difference of 387 bp. This difference was entirely attributable to variation in two non-coding regions. First, the previously reported mitogenome contained a 158 bp intergenic spacer between tRNA-Thr and tRNA-Pro, whereas these two genes overlapped by 1 bp in the present assembly, resulting in a 159 bp difference. Second, the control region length differed between the two mitogenomes, with a 934 bp CR identified in the present HiFi-derived assembly compared with approximately 1.16 kb in the previously published sequence. Together, these two regions explained the complete difference in mitochondrial genome length between the two *P. chungtienensis* records.

Tandem-repeat analysis further identified a repeat-rich region near the 3′ end of the control region in the previously reported *P. chungtienensis* mitogenome, containing a repeat motif of approximately 54 bp. Under the same Tandem Repeats Finder parameters, no comparable tandem-repeat array was detected in the control region of the present HiFi-derived mitogenome. These results indicate that variation in repetitive non-coding regions may contribute substantially to mitochondrial genome length differences among independent mitochondrial genome records.

Overall, comparative analysis of available *Ptychobarbus* mitochondrial genomes demonstrated that mitochondrial gene organization is highly conserved, whereas sequence variation is mainly associated with non-coding regions and repetitive elements. The newly assembled long-read mitogenome provides an additional high-quality mitochondrial resource for comparative studies of *Ptychobarbus* and highlights the importance of evaluating non-coding-region structures when comparing mitochondrial genome records generated using different sequencing strategies.

### 3.3. Mitochondrial Genome-Based Phylogenetic Analysis

**Figure 3:**
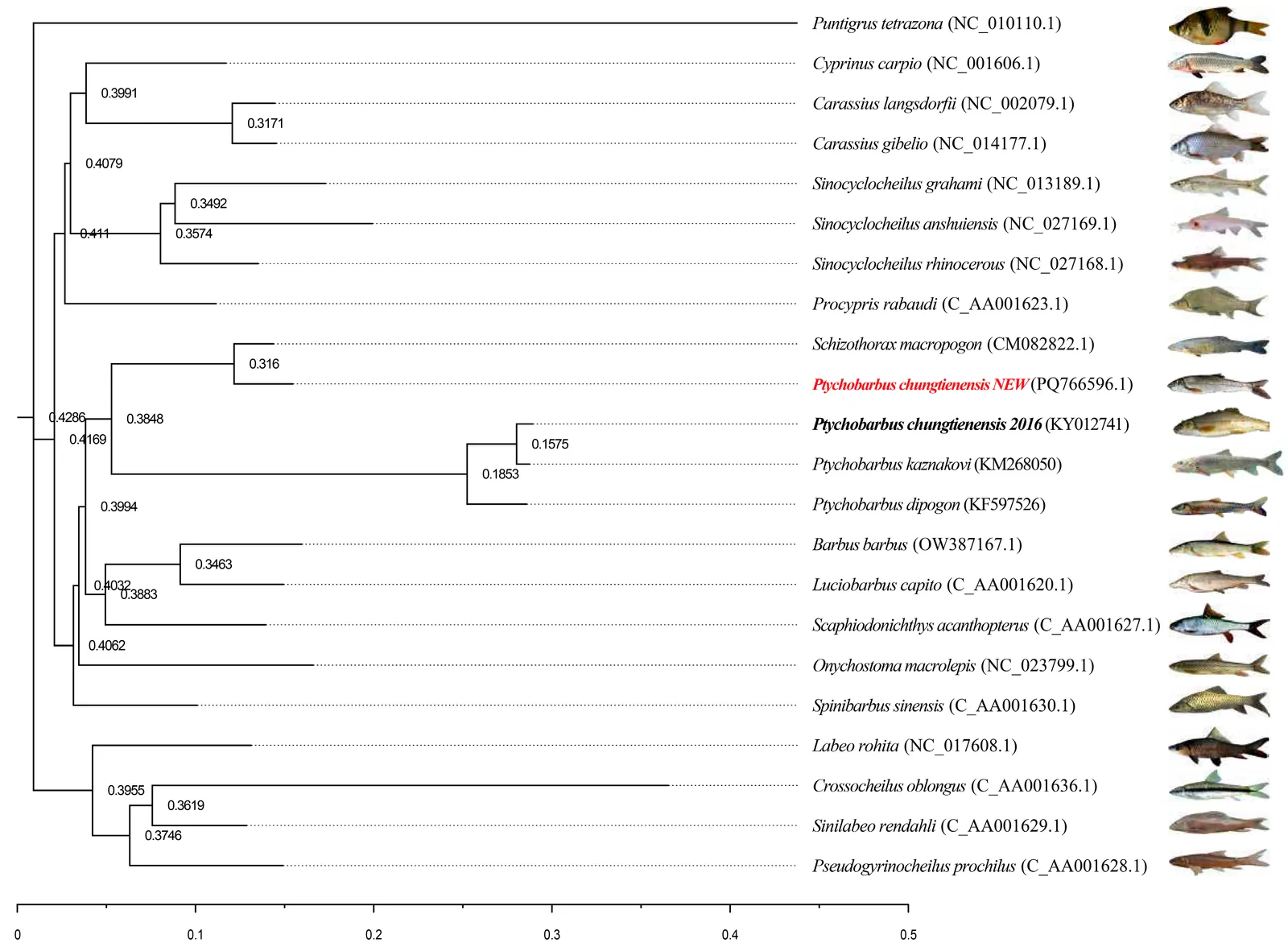
Maximum-likelihood phylogenetic tree based on 22 complete mitochondrial genome sequences of *Ptychobarbus* and related cyprinid fishes. The tree was inferred using IQ-TREE under the TIM2 + F + I + R3 substitution model. Numbers at nodes indicate bootstrap support values (%) from 1000 replicates. *Puntigrus tetrazona* was used as the outgroup. The newly assembled *P. chungtienensis* mitogenome (PQ766596.1) is highlighted (highlighted in red).

The revised maximum-likelihood phylogeny was reconstructed using 22 complete mitochondrial genome sequences, including the newly assembled *P. chungtienensis* mitogenome, the previously reported *P. chungtienensis* sequence, two additional *Ptychobarbus* species (*P. dipogon* and *P. kaznakovi*), and representative cyprinid fishes, with *Puntigrus tetrazona* designated as the outgroup (Figure 3). The phylogenetic analysis was performed using IQ-TREE under the TIM2 + F + I + R3 substitution model, and node support was evaluated using 1000 bootstrap replicates.

The maximum-likelihood tree revealed distinct mitochondrial lineages among the available *Ptychobarbus* records. The newly assembled *P. chungtienensis* mitogenome (PQ766596.1) clustered with *Schizothorax macropogon* with strong bootstrap support (100%). In contrast, the previously published *P. chungtienensis* mitogenome (KY012741.1/NC_034230.1) formed a well-supported clade with *P. kaznakovi* (KM268050.1) (100%), and this clade was further grouped with *P. dipogon* (KF597526.1) with complete bootstrap support (100%). The lineage containing the newly assembled *P. chungtienensis* and *S. macropogon* was subsequently grouped with the clade comprising the previously reported *P. chungtienensis*, *P. kaznakovi*, and *P. dipogon*, with moderate bootstrap support (88%).

The two available mitochondrial genome records of *P. chungtienensis* therefore occupied different positions within the mitochondrial phylogeny based on the current taxon sampling. Several deeper nodes within the phylogenetic tree showed relatively low or moderate bootstrap support, indicating uncertainty in some intergeneric relationships. Because this analysis was based exclusively on mitochondrial genomes, the inferred topology should be interpreted as mitochondrial lineage relationships rather than a definitive representation of species-level evolutionary history. Additional nuclear genomic and population-level evidence will be required to further resolve the evolutionary relationships within *Ptychobarbus*.

### 3.4. Selection Pressure Analysis of Mitochondrial Protein-Coding Genes

Selection-pressure analyses were performed separately for the 13 mitochondrial protein-coding genes (*ND1*, *ND2*, *COX1*, *COX2*, *ATP8*, *ATP6*, *COX3*, *ND3*, *ND4L*, *ND4*, *ND5*, *ND6*, and *CYTB*). Under the one-ratio model (M0), the estimated dN/dS (ω) values ranged from 0.00914 for *COX1* to 0.16481 for *ATP8* (Table 2). All mitochondrial protein-coding genes showed ω values substantially lower than 1, indicating that these genes have been predominantly constrained by purifying selection during mitochondrial genome evolution.

The two-ratio branch model was further applied to evaluate whether the newly sequenced *P. chungtienensis* lineage showed lineage-specific changes in selective pressure. The estimated foreground ω values ranged from 0.00010 for *ND3* to 0.25658 for *ATP8*. Although several genes, including *COX1*, *COX2*, *ATP8*, *ATP6*, *COX3*, *ND4L*, *ND6*, and *CYTB*, exhibited slightly higher foreground ω values than background branches, likelihood-ratio tests showed no significant differences in selective pressure between the foreground and background branches for any of the 13 mitochondrial PCGs (*p* > 0.05).

Branch-site analyses were further conducted to detect potential positive selection affecting specific codon sites along the newly sequenced *P. chungtienensis* lineage. None of the 13 mitochondrial protein-coding genes showed significant evidence of positive selection. The highest likelihood-ratio statistic was observed for *ND4L* (2ΔlnL = 2.635, *p* = 0.105), but this result did not reach statistical significance. Overall, the mitochondrial protein-coding genes analyzed in this study showed strong evolutionary conservation, with no significant evidence of lineage-specific adaptive selection detected along the newly sequenced *P. chungtienensis* branch.

## 4. Discussion

In this study, we assembled and annotated a complete mitochondrial genome of the Chungtien schizothoracin (*Ptychobarbus chungtienensis*) using PacBio HiFi sequencing data. The newly generated mitogenome was 16,583 bp in length and contained the typical vertebrate mitochondrial gene complement, including 13 protein-coding genes (PCGs), 22 tRNA genes, two rRNA genes, a control region (CR), and the origin of light-strand replication (O*_L_*). The overall gene organization, strand distribution, and nucleotide composition were consistent with the conserved mitochondrial genome characteristics generally observed in teleost fishes [10,23]. Most mitochondrial genes were encoded on the heavy strand, whereas *ND6* and eight tRNA genes were located on the light strand. The A + T content of the present mitogenome was 54.97%, with positive AT-skew and negative GC-skew values, reflecting common compositional characteristics of fish mitochondrial genomes. However, these nucleotide composition patterns represent general features of mitochondrial genome evolution and should not be interpreted as direct evidence of environmental adaptation.

A complete mitochondrial genome of *P. chungtienensis* was previously reported by Qiu and Chen using a PCR-based sequencing approach, with a total length of 16,970 bp [1]. The present PacBio HiFi-derived mitogenome provides an independent mitochondrial sequence resource and enables direct comparison of genome structure and sequence characteristics between two independently generated records. The observed 387 bp difference between the two mitochondrial genomes was entirely attributed to variation in non-coding regions. Specifically, the previously reported mitogenome contained a 158 bp intergenic spacer between tRNA-Thr and tRNA-Pro, whereas these two genes overlapped by 1 bp in the present assembly. In addition, the control region of the present mitogenome was 934 bp, whereas the corresponding region in the previously reported sequence was approximately 1.16 kb. Tandem-repeat analysis further revealed a repeat-rich region near the 3′ end of the previously reported control region, containing an approximately 54 bp repeat motif, whereas no comparable tandem-repeat array was detected in the present HiFi-derived sequence. Because mitochondrial control regions are generally variable and frequently contain repetitive elements, differences in repeat organization may contribute substantially to mitochondrial genome length variation among independent records [10,11]. Nevertheless, the present study only characterizes structural differences between mitochondrial sequences, and further experimental validation would be required to determine whether these differences reflect sequencing strategy, biological variation, or other factors.

The revised mitochondrial phylogenetic analysis provided additional insights into the relationships among available *Ptychobarbus* mitochondrial lineages. The newly assembled *P. chungtienensis* mitogenome clustered with *Schizothorax macropogon* with strong bootstrap support, whereas the previously reported *P. chungtienensis* sequence was positioned within a different mitochondrial lineage together with *P. kaznakovi* and *P. dipogon*. These results indicate that the currently available *P. chungtienensis* mitochondrial records occupy different positions in the mitochondrial phylogeny under the present taxon sampling. However, mitochondrial phylogenies represent maternal lineage histories rather than complete species evolutionary histories [5,24]. The observed discordance may result from multiple factors, including mitochondrial lineage variation, historical introgression, incomplete lineage sorting, sampling differences, or unresolved taxonomic issues. Therefore, the present results should not be interpreted as evidence for species-level relationships or taxonomic conclusions. Additional population-level sampling and independent nuclear genomic evidence will be necessary to clarify the evolutionary relationships within *Ptychobarbus* and related schizothoracine fishes.

Selection-pressure analyses further characterized the evolutionary patterns of mitochondrial protein-coding genes in *P. chungtienensis*. All 13 mitochondrial PCGs exhibited dN/dS (ω) values substantially below 1 under the one-ratio model, indicating strong purifying selection acting on mitochondrial protein-coding sequences. Although several genes showed slightly different foreground and background ω estimates in the branch model, none of these differences reached statistical significance. Similarly, branch-site analyses did not detect significant evidence of positive selection along the newly sequenced *P. chungtienensis* lineage. Previous studies have suggested potential associations between mitochondrial genomic variation and high-altitude adaptation in some schizothoracine fishes [5,6]. However, the absence of significant lineage-specific positive selection signals in the present mitochondrial dataset suggests that mitochondrial protein-coding evolution alone does not provide sufficient evidence to explain environmental adaptation in *P. chungtienensis*. Adaptation to plateau environments is likely influenced by complex interactions among nuclear genes, mitochondrial function, physiological regulation, and ecological factors [7,8]. Comprehensive genomic and functional studies will therefore be required to further investigate adaptive mechanisms in this species.

From a conservation perspective, the present study provides an additional mitochondrial genomic resource for *P. chungtienensis*, a threatened freshwater fish with a restricted geographical distribution. Previous studies have highlighted conservation concerns associated with habitat disturbance and the introduction of non-native fishes within its distribution range [2,3]. The newly assembled mitogenome, together with previously available mitochondrial records, provides valuable molecular information for future studies on species identification, mitochondrial diversity, and evolutionary history. However, mitochondrial data alone are insufficient for evaluating population genetic status or conservation priorities. Future studies integrating broader geographic sampling, population-scale mitochondrial variation, nuclear genomic resources, and ecological information will be essential for assessing genetic diversity, population structure, demographic history, and conservation strategies for *P. chungtienensis*.

## 5. Conclusions

In conclusion, this study characterized a 16,583 bp complete mitochondrial genome of *Ptychobarbus chungtienensis* based on PacBio HiFi sequencing, containing the canonical 37 mitochondrial genes, a control region, and the origin of light-strand replication. Comparison with the previously reported 16,970 bp mitogenome revealed pronounced differences in both non-coding-region structure and overall mitochondrial sequence. Phylogenetic analysis further showed that the newly assembled and previously reported *P. chungtienensis* mitogenomes occupied distinct mitochondrial lineages within the current taxon sampling. Selection-pressure analyses of the 13 mitochondrial protein-coding genes detected no significant evidence of lineage-specific positive selection. These results provide an improved mitochondrial genomic resource for comparative mitogenomic and conservation genetic studies of *P. chungtienensis*, while indicating that broader population sampling and independent nuclear genomic evidence will be required to resolve the evolutionary relationships among the divergent mitochondrial lineages.

## Figures and Tables

**Table 1:** Comparative characteristics of complete mitochondrial genomes of *Ptychobarbus*.

Species	Accession	Length (bp)	A + T (%)	AT-Skew	GC-Skew	CR Length (bp)	O*_L_* Length (bp)
*P. chungtienensis* (present study)	PQ766596.1	16,583	54.97	0.08	−0.208	934	33
*P. chungtienensis* (2016)	KY012741.1/NC_034230.1	16,970	54.2	0.026	−0.174	~1162^a^	NR
*P. dipogon*	KF597526.1	16,787	53.76	0.029	−0.173	939	33
*P. kaznakovi*	KM268050.1	16,842	54.3	0.028	−0.177	NR	NR

Note: “a” The original 2016 publication reported a D-loop (control region) length of 1161 bp. The approximately 1162-bp value shown here corresponds to the boundary definition used for direct sequence comparison in the present study. “NR” not reported or not independently confirmed from the available records.

**Table 2:** Selection-pressure analyses of the 13 mitochondrial protein-coding genes.

Gene	M0 ω	Background ω	Foreground ω	Branch-Model LRT	*p* Value	Branch-Site LRT	*p* Value
*ND1*	0.02232	0.02266	0.00749	1.634	0.201	0.000	1.000
*ND2*	0.05559	0.05559	0.05526	<0.001	0.990	0.000	1.000
*COX1*	0.00914	0.00900	0.02107	1.031	0.310	0.000	1.000
*COX2*	0.03785	0.03752	0.06804	0.476	0.490	0.000	1.000
*ATP8*	0.16481	0.16291	0.25658	0.175	0.676	0.000	1.000
*ATP6*	0.02181	0.02175	0.02556	0.021	0.884	0.000	1.000
*COX3*	0.01425	0.01404	0.03785	0.621	0.431	0.000	1.000
*ND3*	0.04741	0.04835	0.00010	2.601	0.107	0.000	1.000
*ND4L*	0.02150	0.02116	0.03766	0.250	0.617	2.635	0.105
*ND4*	0.02443	0.02456	0.01790	0.284	0.594	0.000	1.000
*ND5*	0.04457	0.04472	0.03658	0.171	0.679	0.000	1.000
*ND6*	0.03546	0.03538	0.03948	0.018	0.894	0.000	1.000
*CYTB*	0.02447	0.02445	0.02540	0.003	0.955	0.172	0.678

Note: M0 ω represents the dN/dS ratio estimated under the one-ratio model. Background and foreground ω values were estimated using the two-ratio branch model, with the newly sequenced *P. chungtienensis* lineage designated as the foreground branch. LRT, likelihood-ratio test. No branch-model or branch-site comparison was statistically significant at *p* < 0.05.

## Data Availability

The complete mitochondrial genome assembly data that support the findings of this study are openly available in GenBank of NCBI at (https://www.ncbi.nlm.nih.gov/ (accessed on 13 July 2026)) under the accession no. PQ766596. The associated BioProject, BioSample, and SRA numbers are PRJNA1218292, SAMN46515602, SRR32203064 and SRR32203065, respectively.

## Appendix A

**Table A1:** Whole mitochondrial genomes used to infer the phylogenetic analysis.

Species Name	Size (bp)	Database	Accession Number	URL to Data Record	Reference
*Puntius tetrazona*	16,550	NCBI	NC_010110.1	https://www.ncbi.nlm.nih.gov/nuccore/NC_010110.1 (accessed on 13 July 2026)	Unpublished
*Cyprinus carpio*	16,575		NC_001606.1	https://www.ncbi.nlm.nih.gov/nuccore/NC_001606.1 (accessed on 13 July 2026)	[25]
*Carassius langsdorfii*	16,578		NC_002079.1	https://www.ncbi.nlm.nih.gov/nuccore/NC_002079.1 (accessed on 13 July 2026)	[26]
*Carassius gibelio*	16,581		NC_014177.1	https://www.ncbi.nlm.nih.gov/nuccore/NC_014177.1 (accessed on 13 July 2026)	Unpublished
*Ptychobarbus chungtienensis*	16,583		PQ766596.1	https://www.ncbi.nlm.nih.gov/nuccore/PQ766596.1 (accessed on 13 July 2026)	This study
*Ptychobarbus chungtienensis* (2016)	16,970		KY012741.1	https://www.ncbi.nlm.nih.gov/nuccore/KY012741.1 (accessed on 13 July 2026)	[1]
*Ptychobarbus dipogon*	16,787		KF597526.1	https://www.ncbi.nlm.nih.gov/nuccore/KF597526.1 (accessed on 13 July 2026)	Unpublished
*Ptychobarbus kaznakovi*	16,842		KM268050.1	https://www.ncbi.nlm.nih.gov/nuccore/KM268050.1 (accessed on 13 July 2026)	Unpublished
*Sinocyclocheilus grahami*	16,585		NC_013189.1	https://www.ncbi.nlm.nih.gov/nuccore/NC_013189.1 (accessed on 13 July 2026)	[27]
*Sinocyclocheilus rhinocerous*	16,588		NC_027168.1	https://www.ncbi.nlm.nih.gov/nuccore/NC_027168.1 (accessed on 13 July 2026)	Unpublished
*Schizothorax macropogon*	16,589		CM082822.1	https://www.ncbi.nlm.nih.gov/nuccore/CM082822.1 (accessed on 13 July 2026)	[28]
*Onychostoma macrolepis*	16,595		NC_023799.1	https://www.ncbi.nlm.nih.gov/nuccore/NC_023799.1 (accessed on 13 July 2026)	[29]
*Sinocyclocheilus anshuiensis*	16,618		NC_027169.1	https://www.ncbi.nlm.nih.gov/nuccore/NC_027169.1 (accessed on 13 July 2026)	Unpublished
*Labeo rohita*	16,626		NC_017608.1	https://www.ncbi.nlm.nih.gov/nuccore/NC_017608.1 (accessed on 13 July 2026)	Unpublished
*Barbus barbus*	16,600	CNCB	OW387167.1	https://ngdc.cncb.ac.cn/genbase/search/gb/OW387167.1 (accessed on 13 July 2026)	[30]
*Crossocheilus oblongus*	17,470		C_AA001636.1	https://ngdc.cncb.ac.cn/genbase/search/gb/C_AA001636.1 (accessed on 13 July 2026)	
*Luciobarbus capito*	16,607		C_AA001620.1	https://ngdc.cncb.ac.cn/genbase/search/gb/C_AA001620.1 (accessed on 13 July 2026)	
*Procypris rabaudi*	16,593		C_AA001623.1	https://ngdc.cncb.ac.cn/genbase/search/gb/C_AA001623.1 (accessed on 13 July 2026)	
*Pseudogyrinocheilus prochilus*	16,596		C_AA001628.1	https://ngdc.cncb.ac.cn/genbase/search/gb/C_AA001628.1 (accessed on 13 July 2026)	
*Scaphiodonichthys acanthopterus*	16,594		C_AA001627.1	https://ngdc.cncb.ac.cn/genbase/search/gb/C_AA001627.1 (accessed on 13 July 2026)	
*Sinilabeo rendahli*	16,587		C_AA001629.1	https://ngdc.cncb.ac.cn/genbase/search/gb/C_AA001629.1 (accessed on 13 July 2026)	
*Spinibarbus sinensis*	16,593		C_AA001630.1	https://ngdc.cncb.ac.cn/genbase/search/gb/C_AA001630.1 (accessed on 13 July 2026)	
